# Analgesic Effect of Intra-Articular Injection of Temperature-Responsive Hydrogel Containing Bupivacaine on Osteoarthritic Pain in Rats

**DOI:** 10.1155/2015/812949

**Published:** 2015-12-31

**Authors:** Taemin Kim, Dong Rim Seol, Suk-Chan Hahm, Cheolwoong Ko, Eun-Hye Kim, Keyoungjin Chun, Junesun Kim, Tae-Hong Lim

**Affiliations:** ^1^Department of Physical Therapy, Korea University College of Health Science, Seoul 136-713, Republic of Korea; ^2^Rehabilitation Science Program, Department of Public Health Science, Graduate School, Korea University, Seoul 136-713, Republic of Korea; ^3^Department of Biomedical Engineering, The University of Iowa, Iowa City, IA 52242, USA; ^4^Advanced Biomedical and Welfare Group, Korea Institute of Industrial Technology (KITECH), Chungnam 330-825, Republic of Korea

## Abstract

The present study examined the analgesic effects of slow-releasing bupivacaine from hydrogel on chronic arthritic pain in rats. Osteoarthritis (OA) was induced by monosodium iodoacetate (MIA) injection into the right knee joint. Hydrogel (HG: 20, 30, and 50 *μ*L) and temperature-sensitive hydrogel containing bupivacaine (T-gel: 20, 30, and 50 *μ*L) were injected intra-articularly 14 days after MIA injection. Behavioral tests were conducted. The rats showed a significant decrease in weight load and paw withdrawal threshold (PWT). Intra-articular 0.5% bupivacaine (10 and 20 *μ*L) significantly reversed MIA-induced decreased PWT, with no effect on weight load. In normal rats, hydrogel did not produce significant changes in PWT but at 30 and 50 *μ*L slightly decreased weight bearing; T-gel did not cause any changes in both the weight load and PWT. In OA rats, T-gel at 20 *μ*L had a significant analgesic effect for 2 days, even though T-gel at 50 *μ*L further reduced the weight load, demonstrating that intra-articular T-gel (20 *μ*L) has long-lasting analgesic effects in OA rats. Thus, T-gel designed to deliver analgesics into the joint cavity could be an effective therapeutic tool in the clinical setting.

## 1. Introduction

Osteoarthritis (OA) is the most common chronic disease in the elderly and leads to impairment of mobility and disability [1, 2]. Pain is the most noticeable symptom, and it can affect the quality of life of patients with OA [3]. Inflammation of the synovial membrane in joints produces unexpected painful sensations that are characterized by nonevoked (spontaneous) pain and evoked pain such as hyperalgesia (exaggerated response to painful stimuli) and allodynia (normal innocuous sensory stimuli that are perceived as painful). These sensations result from sensitization of peripheral afferent nerves and dorsal horn neurons [4]. Moreover, adaptive changes in the nervous system caused by long-term inflammation may result in an exaggeration of abnormal sensations of pain.

Despite efforts to manage chronic arthritic pain, medications have limited effects as well as accompanying side effects. For example, acetaminophen or nonsteroidal anti-inflammatory drugs (NSAIDs) are commonly prescribed for OA pain relief and administered either orally or locally [5], but long-term use of NSAIDs can cause side effects such as indigestion, stomach ulcers, and gastrointestinal bleeding. Sometimes a higher dose of the drug is required to control chronic OA pain. However, the maximum bolus of the drug is limited by potential toxicity or severe side effects. Additionally, there exist no ideal drugs or nonsurgical treatment methods for immediate and long-term relief of OA pain and arthritic conditions.

An ideal nonsurgical OA treatment method would consist of a delivery system that can deliver the drug(s) into the knee by percutaneous injection and release the drug(s) over a prolonged period of time. One combination of drugs administered by such a system would be both a local anesthetic (e.g., bupivacaine) for effective pain control and a nonsteroidal analgesic (e.g., naproxen or ibuprofen) for local treatment of inflammation in the joint. Recently, in situ forming reverse thermal hydrogel (HG) consisting of F-127 (BASF, Gurney, IL) and hyaluronic acid (Easy Motion Horse, Niagara Falls, NY) was introduced as a biocompatible and injectable drug delivery vehicle [6, 7]. Lee et al. demonstrated the excellent injectability of HG as well as its slow release of bupivacaine in an* in vitro* test [6]. The biocompatibility and preclinical feasibility of HG containing 0.5% (w/v) bupivacaine for use in pain control after surgical wounds were found to be excellent; an* in vivo* rat study found minimal inflammatory reactions after HG application, with no negative impact with and without 0.5% (w/v) bupivacaine on surgical wound healing [7]. In the same study, the release of bupivacaine from the HG in a controlled manner over 3 or more days was also demonstrated in an* in vitro* drug release test. However, the analgesic effect of HG with bupivacaine over a prolonged period of time was not tested in Seol et al.'s study [7]. Another critical aspect to consider in the use of HG containing bupivacaine would be the possible side effects on the OA knee. Other important factors for developing an ideal nonsurgical method using HG containing both a local anesthetic drug and NSAID include the selection of appropriate drugs, determination of their working dosage, and method of loading the drugs into the HG.

In this study, the feasibility of using bupivacaine containing HG vehicle, first introduced in Seol et al.'s study [7] as a part of a nonsurgical method to treat OA joints, was tested in terms of biocompatibility and efficacy for prolonged pain relief. During this test, the appropriate dose of bupivacaine and the maximum volume of HG possible for safe and effective use were also determined.

## 2. Materials and Methods

### 2.1. Experimental Animals

Adult male Sprague-Dawley rats (180–200 g, *n* = 68; Orient Bio Inc., Seoul, Korea) were used in this study. The animals were acclimated for at least 5 days prior to experiment. The rats were housed in a 12-hour light/dark cycle (08:00–20:00) and were provided free access to standard animal water and food. Guidelines set by the Korea University Institutional Animal Care and Use Committee for the care and use of laboratory animals (KUIACUC-2014-10) have been observed.

### 2.2. Induction of Osteoarthritis

Osteoarthritis was induced by single intra-articular injection of monosodium iodoacetate (MIA, 4 mg/50 *μ*L; Sigma Aldrich, ST. Louis, MO, USA) into the joint cavity of the right knee. Briefly, MIA was injected using a 30-gauge needle through a pathway under the patella tendon and directly into the synovial space of the knee joint, flexed 90°, of a rat under isoflurane anesthesia (2% isoflurane in O_2_). Following injection, the animals were allowed to recover from the effects of anesthesia before they were returned to their home cage (usually 5–7 min). All animals underwent the behavioral tests described below in order to obtain baseline data related to pain in the knee.

### 2.3. Preparation and Administration of Drugs

Saline solution with bupivacaine (0.5% w/v), reverse thermal hydrogel with no bupivacaine (HG), and the HG containing 1.25% (w/v) bupivacaine (T-gel) were prepared in a clean environment according to the method used in Seol et al.'s study [7]. The total dose of bupivacaine (microgram)/total volume (microliter) is 125/10, 250/20, 375/30, or 625/50, which is still 1.25% (w/v). The dose of bupivacaine in T-gel was chosen based on the dosage of a new product EXPAREL (Pacira Pharmaceuticals, Inc.), recently approved by the US FDA for pain control in surgical wounds for up to 72 h. In EXPAREL, 1.3% bupivacaine is slowly released from depofoam microspheres. Cleanliness of HG and T-gel (free of bacteria and toxins) was confirmed by 24 h culture of and toxin test of samples obtained from all batches.

Prepared solutions of various volumes (10, 20, 30, or 50 *μ*L) were administered into the knee joint space 14 days after MIA injection. Normal rats received an injection of HG or T-gel in the knee to test biocompatibility of HG and T-gel and saline (10, 20, 30, or 50 *μ*L) was injected into the knee joint in normal or MIA-induced OA rats that served as the control.

### 2.4. Behavioral Tests of the Development and Relief of Pain in OA Knee

All treated animals underwent two behavioral tests, a weight-bearing capacity test and paw withdrawal threshold test, in order to evaluate the analgesic effect of T-gel as well as pain development due to MIA injection. These behavioral tests were performed at the following time points: 1 day before treatment (injection of MIA, HG, or T-gel); 0.5, 1, 1.5, 2, 3, 4, 6, and 8 h; 1, 2, and 3 days after the treatment.

Weight bearing capacity test: a weight-bearing device [8] was used to quantify the weight-bearing capacity of the hind limb of a rat while freely walking. When the rats walked along a path consisting of 8 plates with force sensors, it is possible to measure the ground reaction force under each foot, indicating the weight supported by the corresponding limb. Using ground reaction force data, the weight-bearing index (WBI) was determined by the following formula: [ipsilateral weight/(ipsilateral weight + contralateral weight)] × 100. Thus, a WBI less than 50 indicates the reduced contribution of the treated leg in bearing weight and increased contribution by the contralateral leg. The WBI decreases in the limb of the inflamed (or painful OA) joint because supporting weight increases the perception of pain, a major symptom of arthritis. The test was repeated two or three times on each rat until at least five time-weight curves were obtained for a given limb.

Paw withdrawal threshold (PWT) test: PWT to the application of a von Frey filament (0.41–5.10 g, Stoelting, Wood Dale, IL, USA) has been used as a measure of mechanical allodynia (hypersensitivity). Briefly, a rat was placed in transparent plastic cage (28 cm × 28 cm × 10 cm) on a metal mesh floor and allowed to move freely for 5 min for behavioral accommodation. Then, the von Frey filament was applied to the plantar surface of the hind foot for 3-4 s while the filament was bent. Application began with the thinnest von Frey filament (a force of 2 g) and continued with a thicker filament until a rapid hind paw withdrawal response occurred or the thickest filament (a force of 5.1 g) was used. The stimuli were presented at an interval of several seconds. The PWT value was determined using the up-down method [9] according to a 50% von Frey threshold method of Dixon [10].

### 2.5. Data Analysis

All measured values of WBI and PWT were expressed as group means ± SEM. The Mann-Whitney test was used to compare the mean WBIs and mean PWTs, not only between ipsilateral and contralateral hind paws within a rat, but also among the control groups (administration of saline with bupivacaine and HG) and experimental groups (administration of T-gel of various volumes). Significant changes in the measured parameters over the follow-up time course after treatment were analyzed by Wilcoxon signed rank test. In all comparisons, *p* value < 0.05 was considered to indicate statistical significance.

## 3. Results

### 3.1. Chronic Arthritic Pain in MIA-Induced OA Rats

**Figure 1:**
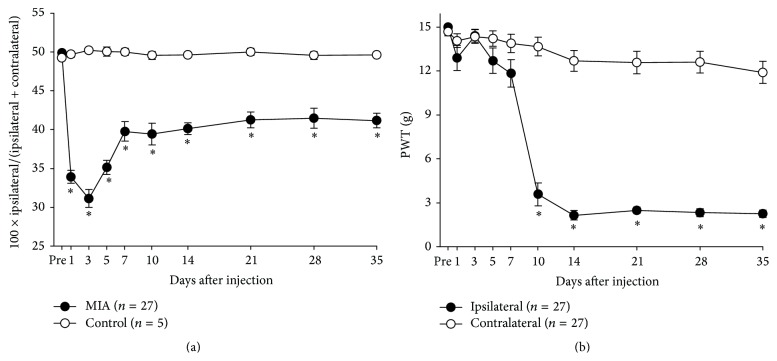
Changes of pain-related behavior after MIA injection into the right knee joint in rats. Behavior tests were performed 1, 3, 5, 7, 10, and 14 days after injection. (a) Weight load was presented by [100 × ipsilateral weight/(ipsilateral + contralateral weight)]. (b) Paw withdrawal threshold (PWT) was measured to test mechanical allodynia. Asterisks indicate a statistically significantly difference from the control group (^*∗*^ *p* < 0.05).

Significant decreases in WBI and PWT values were found in all rats with MIA injection as shown in Figure 1 (*p* < 0.05). MIA injection caused a significant decrease in WBI value from the first day during the follow-up period (Figure 1(a)), with the WBI values remaining significantly below the normal value until 35 days after injection. PWT values measured from the hind paw on the MIA injection side (ipsilateral side) also decreased significantly (Figure 1(b)) over the follow-up period. Significant decrease in PWT was observed from day 10 and persisted until 35 days after MIA injection. Significant decreases in WBI and PWT for 35 days after MIA injection showed successful induction of the painful OA by MIA.

### 3.2. Effects of Bupivacaine on Chronic Arthritic Pain in MIA-Induced OA Rats

**Figure 2:**
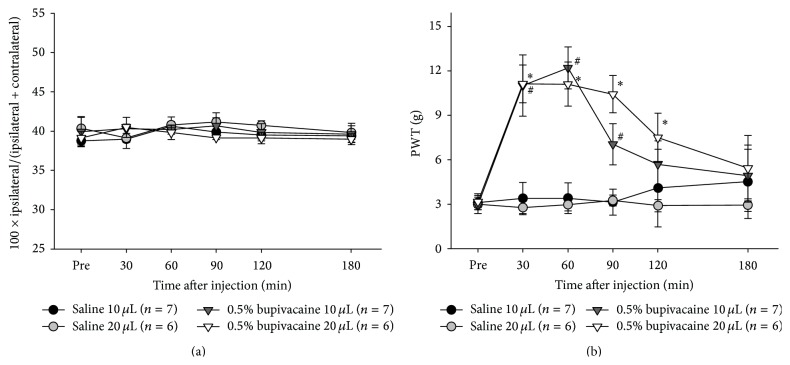
Effects of 0.5% bupivacaine on chronic arthritic pain induced by MIA. (a) Weight load and (b) mechanical allodynia were evaluated before and 30, 60, 90, 120, and 180 min after 0.5% bupivacaine or saline injection into the OA knee joint. Groups marked with asterisks (0.5% bupivacaine 20 *μ*L) or number signs (0.5% bupivacaine 10 *μ*L) indicate a statistically significantly difference from the saline group (^*∗*^ *p* < 0.05, ^#^ *p* < 0.05).

To investigate the effects of bupivacaine on chronic arthritic pain, 0.5% bupivacaine (10 and 20 *μ*L) was administered into the knee joint 14 days after MIA injection. No changes in WBI from the baseline value were observed for 3 h after intra-articular injection of 0.5% bupivacaine solution into the OA knee, regardless of its volume (Figure 2(a)). In contrast, significant increases in PWT values from baseline were observed after injection of the bupivacaine solution, demonstrative of the significant analgesic effect of bupivacaine (*p* < 0.05). Peak effect was observed at 30–60 min after bupivacaine injection of both 10 and 20 *μ*L volumes. However, injection of 10 *μ*L bupivacaine solution resulted in a significantly increased PWT up to 90 min after injection (Figure 2(b), gray dots). The analgesic effect of 20 *μ*L bupivacaine (Figure 2(b), dark gray dots) was sustained up to 120 min after injection (Figure 2(b)). No significant changes in both WBI and PWT were observed after injection of 10 and 20 *μ*L saline throughout the test period (Figure 2).

### 3.3. Biocompatibility of HG and T-Gel

**Figure 3:**
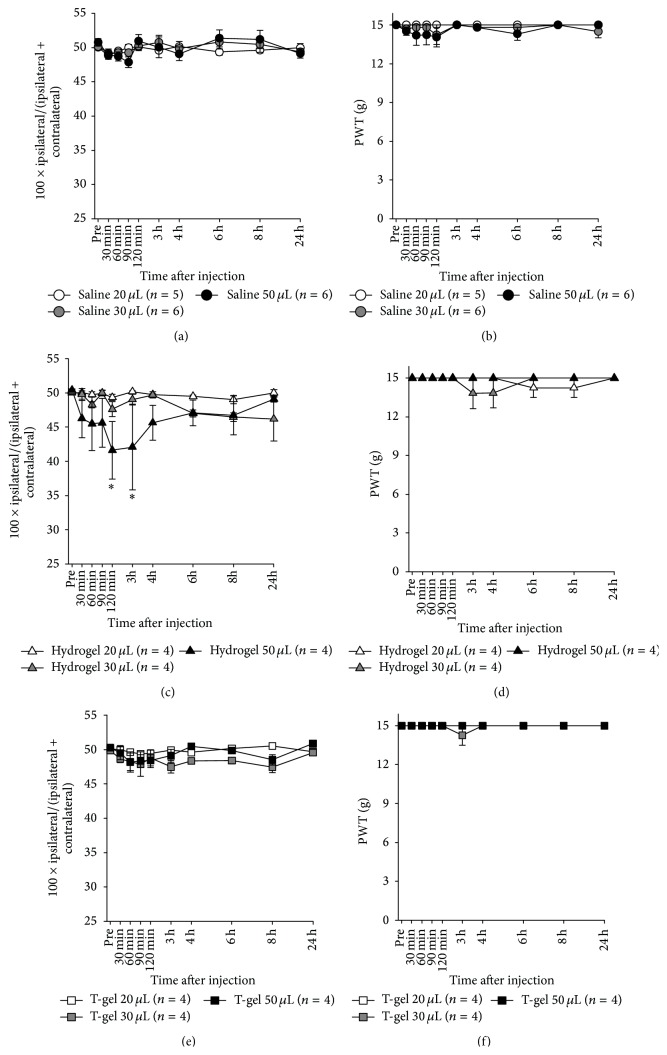
Effects of intra-articular hydrogel or T-gel in normal rats. Weight load (a) and paw withdrawal threshold (PWT) (b) after saline injection. Weight load (c) and PWT (d) after hydrogel injection. Weight load (e) and PWT (f) after T-gel injection. Asterisks indicate a statistically significantly difference from the prevalue (^*∗*^ *p* < 0.05).

To test* in vivo* biocompatibility, various volumes of HG or T-gel were injected into the intact knee joint of normal rats, and WBI and PWT values were measured for 24 h after injection. As shown in Figure 3(c), the injection of 20 or 30 *μ*L HG produced no significant changes in WBI, whereas the injection of 50 *μ*L HG significantly decreased WBI at 2 and 3 h. However, the injection of 50 *μ*L saline into the knee joint of normal rats did not produce any changes in WBI throughout the test period. This result indicates that rats injected with 50 *μ*L HG experienced discomfort in the knee severe enough to result in a significant disturbance in weight-bearing capacity of the knee during walking, but this disturbance lasted only for a couple of hours. In contrast, the injected various volumes of HG did not produce significant changes in PWT values (Figure 3(d)). Figures 3(e) and 3(f) showed no changes in WBI and PWT values, respectively, after injecting various volumes of T-gel. These results clearly demonstrate that both HG and T-gel are highly biocompatible.

### 3.4. Analgesic Effect of HG

**Figure 4:**
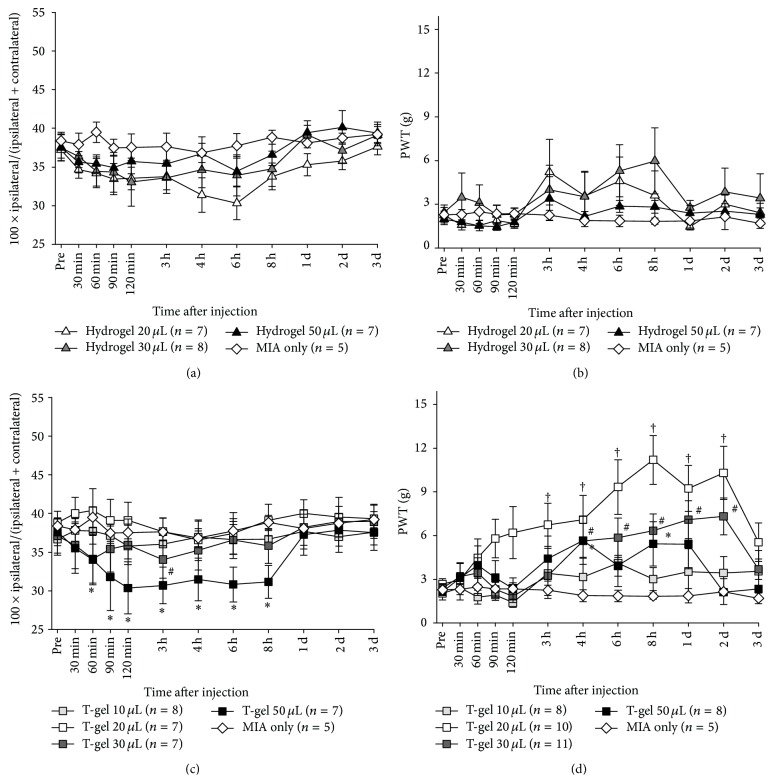
Analgesic effects of intra-articular T-gel on chronic arthritic pain in MIA-induced OA rats. (a) Weight load after hydrogel injection, (b) paw withdrawal threshold (PWT) after hydrogel injection, (c) weight load after T-gel injection, and (d) PWT after T-gel injection. Asterisks (T-gel 50 *μ*L), number signs (T-gel 30 *μ*L), and dagger signs (T-gel 20 *μ*L) indicate a statistically significantly difference from the control group (^*∗*^ *p* < 0.05, ^#^ *p* < 0.05, and ^†^ *p* < 0.05).

HG injected into the knee joint becomes a hydrophilic viscous gel that can provide substantial lubrication to an OA knee and thus relieves pain. The injection of 20, 30, or 50 *μ*L HG into MIA-induced OA knee, however, was found to produce no analgesic effect, as there were no significant changes in both WBI and PWT (Figures 4(a) and 4(b)). These results indicate that HG had neither analgesic nor adverse effect on the OA knee.

### 3.5. Analgesic Effect of T-Gel

The analgesic effect of T-gel was assessed by changes in WBI and PWT values measured in MIA-induced OA rats. T-gel of different volumes (10, 20, 30, or 50 *μ*L) was intra-articularly injected into the painful knee. The injection of 10 *μ*L T-gel produced no changes in WBI (Figure 4(c)) and PWT (Figure 4(d)) when compared to the MIA only sham control (Figures 4(c) and 4(d)), indicating no analgesic effect. In contrast, significant increases in PWT values, shown in Figure 4(d), were found from 3-4 h to 2 days after the intra-articular injection of T-gel (20 or 30 *μ*L) into the painful knee (*p* < 0.05), while there were no changes in WBI values (Figure 4(c)) for 3 days after injection (*p* < 0.05). The increase in PWT value was significant 3 h after 20 *μ*L T-gel injection, but a significant increase was observed 4 h after the 30 *μ*L injection. After injecting 50 *μ*L T-gel, WBI values decreased significantly for the first 8 h but were restored to the initial control value in one day when compared to the MIA only sham control (Figure 4(c)), while PWT values increased significantly for a period from 4 h to one day after injection (Figure 4(d)). In the 50 *μ*L T-gel group, some rats displayed that they walked while lifting up their hindlimb to avoid pressure applied to the joint during the test and the robust reduction of weight load was observed in the injected leg. These results indicate the significant but dose-dependent analgesic effects of T-gel for a period longer than 2 days after injection.

## 4. Discussion

The biocompatibility and prolonged analgesic effect of T-gel were investigated in this* in vivo* study using normal rats and rats with arthritic pain induced by MIA (OA rats).

Various methods such as meniscectomy [11], ligament damage [12], collagenase injection [13], chymopapain injection [14], contusion injury [15], and monosodium iodoacetate (MIA) injection [16] have been used to induce OA in experimental animal studies. Among these experimental models, the MIA-induced OA model is known to most closely mimic the characteristics of the disease in humans because MIA induces chondrocyte necrosis by disturbing glycolysis within the cell [17] and inhibiting proteoglycan synthesis [18]. Recently, the MIA-induced OA rat model has been commonly used to investigate the pathophysiology of OA or the mechanism of osteoarthritic pain, such as in the present study.

Successful induction of painful OA in the knee was confirmed by (1) significant swelling of the knee in terms of an increase in lateral-medial diameter (not reported in this study), (2) significant decreases in weight-bearing capacity of the knee joint in terms of WBI, and (3) development of mechanical allodynia (decrease in mechanical withdrawal threshold). Figures 1(a) and 1(b) demonstrate that painful OA was fully developed in the knee 14 days after MIA injection. Thus, according to these results, the treatment (injection of bupivacaine solution, HG, or T-gel) was administered at least 14 days after MIA injection throughout this study.

The administration of 0.5% bupivacaine (10 or 20 *μ*L) into the painful OA knee produced a significant increases in PWT values from the baseline without any changes in WBI (Figure 2), suggesting that bupivacaine successfully reduced chronic arthritic pain in OA rats. Furthermore, significantly greater PWT values were observed during a 2.5 h period in case of the 20 *μ*L injection, whereas in the case of the 10 *μ*L injection, significantly greater PWTs were observed only during a 1 h period (Figure 2(b)). These results clearly indicate bupivacaine's significant analgesic effect, with the duration varying depending on the amount of bupivacaine injected into the knee. This demonstrates that the MIA-induced rat OA model is an effective model for the purpose of this study.

The T-gel tested in this study contained 1.25% (w/v) bupivacaine. The amount was selected based on the results of other studies. Richard et al. [19] found no evidence of local or systemic toxicities and the absence of histopathologic signs suggesting impairment of the reparative process of wound healing or immune competence of the treated animals when 1.3% bupivacaine was administered in surgical wounds using biodegradable depofoam microspheric delivery vehicles. Seol et al. [7] found that bupivacaine containing HG had an excellent biocompatibility when injected into a subcutaneous region or onto a surgical wound site.

Injection of HG of various volumes into the knee joint did not produce any visual signs of a possible adverse effect, such as weight loss and less movement, over the entirety of the testing periods. The injection of 20 or 30 *μ*L HG into the normal knee was found to produce no changes in WBI and PWI from baseline values. However, the injection of 50 *μ*L HG produced significant decrease in WBI for about 4 h after injection, although no significant decrease in PWT occurred (Figures 3(a) and 3(b)). Sometimes, rat in the 50 *μ*L HG groups displayed a gait with short stance phase of the ipsilateral hindlimb to reduce pressure applied to the joint during the test. In contrast, the injection of T-gel into the normal knee produced no changes in WBI and PWT regardless of the injected volume (Figures 3(c) and 3(d)). These results demonstrated an excellent biocompatibility for HG and T-gel. The injection of HG or T-gel of an excessive volume (>30 *μ*L), however, would increase intra-articular pressure and cause certain discomfort, which would be large enough to make the animal avoid the weight.

The injection of HG into MIA-induced OA rats produced no significant changes in both WBI and PWT (Figures 4(a) and 4(b)), indicating that HG produced no remedial effect. In contrast, T-gel (20, 30, and 50 *μ*L) injected into the arthritic knee joint cavity following MIA injection significantly increased PWT values (Figure 4(d)) but produced no changes in WBI values except for injection of 50 *μ*L T-gel, which produced a significant decrease in WBI value with increased PWT (Figure 4(c)). This disparity of results in the 50 *μ*L T-gel between WBI and PWT can be explained that MIA-induced OA rats tried to reduce the weight in ipsilateral hindlimb due to the excessive pain by joint. Rats with 50 *μ*L HG showed that they could not put weight on their ipsilateral hindlimb and had to hop to avoid pressure applied to the joint during the test. During the mechanical sensitivity test to measure PWT, rats stood or sat with weigh load in their contralateral hindlimb and sometimes their hindlimb was raised by a given force of von Frey filament.

These results clearly demonstrate the analgesic effect of 20 *μ*L T-gel. In terms of bupivacaine dose, the amount of bupivacaine 125 *μ*g in 10 *μ*L T-gel is not sufficient to achieve an analgesic effect, whereas that in 20 *μ*L T-gel containing 250 *μ*g bupivacaine is sufficient for at least 2 days. Considering this, 50 *μ*L T-gel (the total dose of bupivacaine; 625 *μ*g) should release a greater amount of bupivacaine to the region than 20 or 30 *μ*L T-gel (the total dose of bupivacaine; 250 and 375 *μ*g, resp.). However, the analgesic effect of 50 *μ*L T-gel was lower than that of 20 or 30 *μ*L T-gel and had a significant negative effect on WBI, but not 50 *μ*L saline group (Figure 4(d)). It suggests that the concentration of a drug in T-gel should be increased to an effective dose. It should be noted that it is not ideal to use a higher volume of T-gel with a lower drug concentration to deliver the sufficient amount of a therapeutic agent when administering T-gel to a closed space such as the knee cavity, even though our result demonstrated an excellent biocompatibility for HG and T-gel in normal rats.

In the present study, analgesic effect of HG with bupivacaine comes mainly from bupivacaine. Hyaluronic acid (HA) could also have a potential for therapeutic effect on knee OA. HA which is one of components in our HG has been widely used for persistent effective therapy of knee OA [20]. This HA as a viscosupplement enhances viscosity and elastic nature of synovial fluid and finally works as a lubricant. Number of monocytes and fibrotic tissue area in the synovial tissue were minimal close to the uninjured tissue. This study and our results indicate that active ingredients show pharmacological effect while HG works as a vehicle.

Bupivacaine is an amide local anesthetics and mainly used for surgical, obstetric, and pain therapy [21, 22]. Amide type local anesthetics such as bupivacaine bind to intracellular portion of voltage gated sodium channel and then block sodium flux into the cell, which prevents depolarization [22, 23]. Even though clinical report showed that local bupivacaine alleviated pain, the clinical significance was also questioned because of its low analgesic efficacy and relative short duration [24]. Therefore, our aim in this paper was to observe the efficacy and effective duration of HG with bupivacaine, which is designed to release bupivacaine slowly and to compare them based on bupivacaine injection group. Recent advances in biomaterial sciences and technology have led to the development of novel drug delivery systems. In particular, hydrogel, a material that is both injectable and degradable, is one of the most commonly used delivery vehicles in controlled drug delivery systems because of its unique physical properties [25]. Such hydrogel-based systems have been tested for the delivery of not only drugs (chemical compound) but also therapeutic biologic agents. For example, Inoue et al. showed an increase in proteoglycan core protein and type II collagen expression by sustained release of basic fibroblast growth factor (bFGF) from the hydrogel injected into the rabbit knee [26]. Another study demonstrated slow release of dexamethasone from tyramine-modified hyaluronic acid (HA-Tyr) hydrogels for 30 days* in vitro* [27]. Seol et al. [7] described the biocompatibility and feasibility of a reverse thermal in situ forming hydrogel for various applications. They demonstrated the slow release of a drug (bupivacaine) from hydrogel* in vitro* drug release tests, as well as the feasibility of stem cell delivery using an* in vivo* rat study. Their hydrogel consisted of F-127 and hyaluronic acid (HA) and was used as a possible vehicle to deliver and release bupivacaine slowly into the painful OA joint for prolonged pain relief. The results of the present study demonstrated the analgesic effect of bupivacaine, which was released slowly from the hydrogel for almost 3 days. However, the biocompatibility and efficacy of T-gel found in the present study indicates that T-gel needs to be modified for its use in nonsurgical treatment of the arthritic joint because it provides only temporary pain relief (shorter than 3 days), without treating the inflammation associated with OA. One possible method to correct for this would be to incorporate other therapeutic agents to T-gel, such an NSAID, acetaminophen, growth factors, or stem cells. Further studies are required to develop better nonsurgical methods to treat the OA joints.

In addition, the rate of drug release from T-gel, with significant pain relief starting at 3 h after injection and lasting at least 2 days, demonstrates its potential use for other clinical applications, such as local nerve blocks and pain control at the surgical wound site for up to 3 days. A safe and effective dose of bupivacaine (1.3% wt/v) for reliable surgical wound pain control has been already been confirmed in the successful clinical use of EXPAREL. T-gel also can be administered by a single intraoperative injection immediately prior to and/or after closing the surgical openings, such as in the case of EXPAREL. As such, T-gel (HG containing 1.25% bupivacaine) has great potential in the clinical setting as a single injection for the relief of local pain safely and effectively for a few days.

## 5. Conclusions

The findings of this study clearly demonstrate T-gel's prolonged analgesic effect in a dose-dependent manner. More specifically, 20 *μ*L T-gel (HG containing 1.25% bupivacaine) was found to be the most optimal in obtaining prolonged (longer than 2 days) analgesic effect in the OA knee of the rat. While HG has been found to have great potential as a delivery vehicle for diverse therapeutic agents in not only this study but also previous studies [6, 7], the use of a single administration of HG with 1.25% bupivacaine to relieve local pain for almost 3 days is especially promising. Optimum HG volume and dose of therapeutic agent should vary depending upon the site of administration and the type of disease; careful preclinical and clinical studies are needed prior to actual clinical application.
